# The Importance of the Vegetable Course

**Published:** 1918-02-23

**Authors:** 


### FOOD IN WAR-TIME.

### The Importance of the Vegetable Course.

The present emergency presents a fine opportunity for mixing brains with the other ingredients necessary for a good dinner. The Food Controller has directed us to the kitchen garden for the alternative dishes, and it is in this direction that the cook can win her triumphs and incidentally help to win victory for the nation. The vegetable course must be elevated into a position of far greater importance than it has ever held before in English cooking, and those who "can't bear" this, that, and the other vegetable must learn to change their minds and enjoy all that comes.

In the first place, some part of the bread ration must be kept in hand in the form of flour. This will enable the cook to serve the invaluable and easily prepared potato cake for tea, of which the only defect is that it is so appetising when newly baked as to stimulate hunger rather than satisfy it. For breakfast porridge, one ounce of oatmeal will be needed; for supper some thickening ingredient in the soup on the scale of half an ounce a head must be provided. Middle-day dinner then must depend upon meat or fish, vegetables and fruit.

Fortunately, a well-made savoury is far more popular with many people than the ordinary pudding. It takes more trouble to prepare it than the familiar suety or milky pudding, but with a shortage of cereals, of sugar, and of suet, not to mention milk, its value is at once apparent. Pepper, salt, onions, and herbs are still plentiful, and with judicious blending they rival all the sugar in the world as a stimulus to appetite.

Baked cabbage and cheese is a good example of a nourishing savoury containing no rationed ingredients save the modicum of flour needed to blend the sauce. Other excellent and satisfying savouries are scalloped parsnips or potatoes, turnip croquettes, and cauliflowers *au gratin*. A simple savoury consists of sliced beet flavoured with a little vinegar. Eaten with cheese and a slice of bread it presents the maximum of food value. The cook must not be afraid in these day of monotony. Whatever is in season must frequently appear, and the changes must be rung on a comparatively small list of viands. The important thing is to have two or three dishes which are popular, and when this has once been achieved they can be repeated *ad lib.* With the meat or fish course a substantial dish of potatoes (two each at least should be provided) must be supported by vegetables more simply cooked than in the savoury course. Carrots and turnips mashed together are good and satisfying. Turnips are also very good when mashed with potatoes, and so are Jerusalem artichokes. And all are improved by having stewed celery or onion chopped and added to them. Nearly all vegetables are improved by being cooked in stock. Nearly all are savoury and popular when fried, especially if a little bacon fat is available. It is better to put the cook on her mettle in working out such variations than to rely always on formal recipes.

Ripe fruit is seldom seen on the institution table. But nothing is more acceptable or satisfying than a raw apple after a light meal. Used in this way the apple represents better food value than when baked, in which process it sometimes almost disappears. The same is true of lettuces, celery, and other crisp vegetables. In the form of salad they satisfy hunger, while boiled in plain water they make but a dolorous dish.

The following recipes are from Mrs. Lionel Guest's, shilling manual, "Patriotism and Plenty."

### Baked Cabbage and Cheese.

Boil the cabbage, drain thoroughly, and let it get cold. Make a sauce by mixing a tablespoonful of margarine with one of flour, and cooking them together until they bubble; stir in a cupful of milk until thoroughly blended, and season with pepper and salt. Now take a baking-dish, grease it well and sprinkle with pepper and salt. Place in it a layer of the cold cabbage, pour some of the sauce over it, and sprinkle a heaped tablespoonful of grated cheese on the top. Repeat this process till the dish is full, and add a layer of fine breadcrumbs (or mashed potato) on the top. Bake covered for about half an hour, and then remove the cover till the top is browned. This dish is practically a meal in itself, and makes a most delicious course for a dinner.

### Scalloped Parsnips.

Boil some parsnips in boiling salted water and cut them into small dice. Grease a fire-proof baking-dish and cover the bottom with soup stock. Add a layer of the parsnips, a little chopped onion, and a sprinkle of pepper and salt. Now put another layer of stock, and repeat until the dish is full, the last layer being stock. Cover the top with breadcrumbs (or mashed potato), and bake in a hot oven till brown. Cheese may be substituted for the onion if preferred.

### Turnip Croquettes.

Wash, pare, and cut into quarters some new French turnips, strain them till tender, press out all the water possible, and mash. This is most easily done by wringing them in a clean cheese-cloth. Season with pepper and salt, and add the yolks of two eggs well beaten. When the mixture is cold, dip in crumbs, raw egg (or milk), and crumbs again, fry to a rich brown and drain.

